# PSHG-TISS: A collection of polarization-resolved second harmonic generation microscopy images of fixed tissues

**DOI:** 10.1038/s41597-022-01477-1

**Published:** 2022-07-02

**Authors:** Radu Hristu, Stefan G. Stanciu, Adrian Dumitru, Lucian G. Eftimie, Bogdan Paun, Denis E. Tranca, Pavel Gheorghita, Mariana Costache, George A. Stanciu

**Affiliations:** 1 Center for Microscopy-Microanalysis and Information Processing, University Politehnica of Bucharest, 060042 Bucharest, Romania; 2 Department of Pathology, Carol Davila University of Medicine and Pharmacy, Bucharest, Romania; 3 Department of Pathology, Emergency University Hospital, Bucharest, Romania; 4 Department of Pathology, Central University Emergency Military Hospital, Bucharest, Romania; 5 Faculty of Automation and Computer Science, Technical University of Cluj-Napoca, 40002 Cluj-Napoca, Romania; 6 Faculty of Energetics, University Politehnica of Bucharest, Bucharest, Romania

**Keywords:** Cancer imaging, Optical imaging

## Abstract

Second harmonic generation (SHG) microscopy is acknowledged as an established imaging technique capable to provide information on the collagen architecture in tissues that is highly valuable for the diagnostics of various pathologies. The polarization-resolved extension of SHG (PSHG) microscopy, together with associated image processing methods, retrieves extensive image sets under different input polarization settings, which are not fully exploited in clinical settings. To facilitate this, we introduce PSHG-TISS, a collection of PSHG images, accompanied by additional computationally generated images which can be used to complement the subjective qualitative analysis of SHG images. These latter have been calculated using the single-axis molecule model for collagen and provide 2D representations of different specific PSHG parameters known to account for the collagen structure and distribution. PSHG-TISS can aid refining existing PSHG image analysis methods, while also supporting the development of novel image processing and analysis methods capable to extract meaningful quantitative data from the raw PSHG image sets. PSHG-TISS can facilitate the breadth and widespread of PSHG applications in tissue analysis and diagnostics.

## Abstract (summary)

**Table:** 

Measurement(s)	Type I Collagen
Technology Type(s)	multi-photon laser scanning microscopy
Factor Type(s)	second order susceptibility tensor elements
Sample Characteristic - Organism	Homo sapiens
Sample Characteristic - Environment	laboratory environment
Sample Characteristic - Location	Romania

## Background & Summary

Second harmonic generation (SHG)^1^ is a coherent second order nonlinear effect in which two photons are effectively combined via virtual energy states upon the interaction with non-centrosymmetric media. Thus, the result is a new photon with exactly double the energy of any of the two initial photons. Using this process as a contrast mechanism, SHG microscopy has become established as a powerful biomedical investigation technique, enabling to date a wide variety of biological and biophysical imaging applications^2^. Tubulin^3^, myosin^4^ and collagen^5,6^ are known to generate SHG signals, with the latter being one of the most frequently analysed tissue constituents by SHG microscopy. The coherent nature of the SH signals is exploited by polarization-resolved SHG (PSHG) microscopy, which complements intensity-based SHG microscopy. Under varying input laser beam polarization this nonlinear microscopy technique can probe the structure and organization of the harmonophores in the focal volume^5^. The quantitative capabilities of PSHG microscopy are well emphasized by using theoretical models for collagen^7,8^ in combination with various fitting algorithms which return the distribution and organization of collagen^9^ at pixel-level.

Another nonlinear optical effect which is exploited in laser scanning microscopy alongside SHG is two photon excited fluorescence (TPEF). While SHG is a coherent process, TPEF involves the simultaneous absorption of two photons with a total energy enough to produce a transition to an excited state^10^. The two-photon absorption process is subsequently followed by the spontaneous emission of a photon with a slightly smaller energy than the total energy of the two excitation photons. There are minimal or no modification made to an SHG microscope to acquire TPEF signals, hence TPEF microscopy can offer complementary information to SHG microscopy. TPEF allows the non-invasive assessment of cells and tissue morphology^11–13^, alongside the collagen structural characterization provided by SHG.

The results reported throughout the past two decades by using (P)SHG microscopy have demonstrated that the SHG contrast mechanism provides valuable insights into the collagen architecture for different organs, especially for pathologies which are accompanied by specific modifications of the collagen network within the affected tissues. These features of SHG microscopy allowed its use for different pathologies of the skin^14^, breast^15,16^, or thyroid^17,18^, without being limited just to imaging, but also extending its application to quantitative analyses of the collagen distribution.

Various experiments reported to date showed that a wide variety of image features can be extracted from SHG images to quantitatively characterize collagen in tissue samples. The SHG directional emission ratio (forward SHG to backward SHG ratio) was shown to be correlated with the fibril diameter and the collagen density^19,20^. Fractal analysis^18,21^ was used to extract collagen-related morphological data from SHG images, while whole image Fast Fourier Transform^22,23^ and wavelet transform^24^ were able to quantify the texture and orientation changes of collagen. Inter-pixel relationship in SHG images were quantified by using texture analysis in which second-order image statistics are evaluated from the gray-level co-occurrence matrix (GLCM)^9,18,25^. On the other hand, PSHG image sets retrieved information on the collagen orientation and helical pitch angle^26^, the elements of the second order nonlinear susceptibility tensor (χ^(2)^) for collagen^9,27^ and the fitting efficiency^28^. Other aspects reflecting the importance of specific PSHG image features for quantitative tissue analysis are nicely summarized in the recent review of R. Cisek *et al*.^29^.

At the same time with SHG bioimaging gaining more and more interest, over the past years the field of computer vision has also significantly expanded. These scientific domains have successfully intersected, and valuable methods aimed at the automated classification of different tissue types and pathologies have been reported to date. By using machine learning (ML) techniques dealing with various SHG image features, such as those previously discussed, a consistent number of applications were proposed. Among others, such efforts enabled the accurate classification of SHG images on different ovary pathologies^30^, in human idiopathic pulmonary fibrosis^24^ or in the liver fibrosis^31^. Unlike ML techniques, training of deep learning (DL) classifiers requires little or no intervention. Moreover, DL is known for its superb transfer learning capabilities, a feature that allows neural networks to be trained on one sort of data and applied on a different data type, e.g., training on natural images and testing on biomedical images^32^. Scoring liver fibrosis^33^, identifying dysplastic skin tissue^12^ or predicting the elasticity of collagenous tissue^34^ are just a few examples where DL was used in conjunction with SHG imaging. One of the important bottlenecks in using DL in combination with SHG images (and with advanced microscopies in general), is the limited training data availability, as insufficient training data is known to take a significant toll on a DL method’s ability to generalize.

**Fig. 1:**
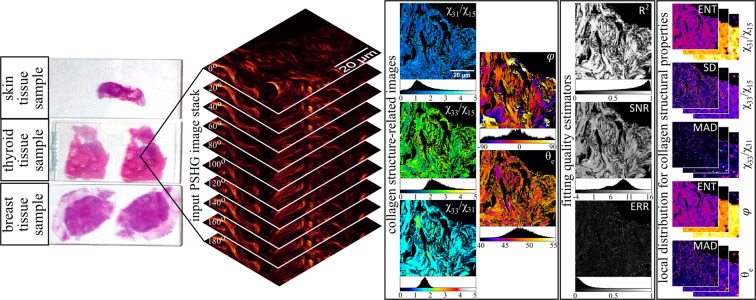
Schematic overview of the protocol of generating the images from the initial PSHG image stack. The PSHG image stack is used to generate five collagen structure-related images with three additional fitting quality estimator images. Using entropy (ENT), standard deviation (SD) and median absolute deviation (MAD), the local distribution for each of the five collagen structural properties can be estimated in six computational windows (square and “circular” each with three different sizes).

The proposed image collection, PSHG-TISS, relies on PSHG image stacks collected on three different tissue types (breast, skin, thyroid), with a PSHG stack consisting of SHG images acquired at varying input polarization angles of the excitation laser beam. Besides providing the raw PSHG image stacks, we also include computed representations (Fig. 1) that depict the 2D distribution of various parameters known to be important in the evaluation of the collagen structure (i.e., χ^(2)^ tensor elements ratios, the collagen orientation and the helical pitch angle^26^ for collagen) as well as parameters which can be used for selecting relevant pixels in the images (i.e., the coefficient of determination, a measure of error of the fitting algorithm and signal-to-noise ratio). Furthermore, this image set was extended by computing images that show the local distribution of the parameters for the collagen structure using two measures of dispersion (standard deviation and median absolute deviation) and local entropy. For each of the available PSHG sets we also provide a TPEF image which can be used to place the collagen imaged by SHG microscopy in a tissular context, since cells are perfect sources for TPEF. We envision that the proposed image collection can be useful for the development and benchmarking of (P)SHG image processing and analysis methods or in DL approaches for image sets augmentation or transfer learning.

## Methods

### Sample preparation

The proposed image set includes PSHG images acquired on breast, skin, and thyroid tissue samples. The use of the imaged tissue samples for scientific research purposes was approved by the Emergency University Hospital, Bucharest, Romania (breast and skin tissue samples) and by the Carol Davila University Central Emergency Military Hospital, Bucharest, Romania (thyroid tissue samples). Written informed consent was obtained from the patients and all samples were deidentified prior to imaging. All methods were performed according to relevant guidelines and regulations and in accordance with the Declaration of Helsinki. The tissue samples were processed according to standard histology procedures which are presented in the following:

1. Tissue fixation which prevents tissue alteration and autolysis. It is made by immersing the tissue fragments in 10% formalin for one hour in the first bath and two hours in the second.
2. Dehydration removes the residual fixative and the water within cells. It was achieved with alcohols with gradually increasing concentration (70%, 80%, 96% and 100%), with one hour per stage.
3. Clearing consists in removing the alcohol from the tissue fragment using a paraffin solvent (i.e., toluene). It also dissolves lipids and allow for a complete wax infiltration. For this stage, three solvent baths are used, the duration for each being one hour.
4. Paraffin inclusion is the process in which the tissue fragments are impregnated with paraffin wax to support the tissue for thin sectioning, resulting a paraffin-embedded tissue block.
5. Microtomy is used to obtain thin tissue sections (2–4 μm) from the paraffin-embedded tissue blocks. The thin tissue sections floating on a warm water bath to remove wrinkles are finally placed on the glass slide.
6. Deparaffination stage removes the wax by hot drying the sections for 20–30 minutes. The tissue sections are further placed in heated toluene for another 20–30 minutes.
7. Hydration enables the staining with the aqueous hematoxylin solution. To do this the tissue sections are placed in alcohol of decreasing concentration (100%, 96%, 80%, 70%), with each stage taking 5 minutes and finally in water.
8. Hematoxylin and Eosin (H&E) staining. Nuclei are stained blue in hematoxylin for 5 minutes. The tissue section is washed in two tap water baths and placed in the third for differentiation. The tissue section is then passed in Eosin for 5 minutes and finally washed in 1–3 water baths.
9. Dehydration in increasing concentration of alcohols (70%, 80%, 96%, 100%), with each stage taking 5 minutes.
10. Clearing into toluene for 5–15 minutes.
11. Mounting with a low viscosity xylene-based medium for permanent covering of microscope slides and covering with glass coverslips.

The H&E-stained samples followed the entire processing protocol, while additional samples were left unstained (steps 7 and 8 were skipped). Regions of interest (ROIs) were selected by trained pathologists for imaging with the PSHG microscope.

### Imaging setup

The imaging of the thin tissue sections was performed with a three-channel Leica TCS SP laser scanning confocal microscope adapted for nonlinear imaging. The excitation source was a Ti:Sapphire laser (Coherent Chameleon Ultra II) emitting at 870 nm with pulse widths of 140 fs and a repetition rate of 80 MHz. Laser beam power levels lower than 15 mW, measured in the objective focus, were used during scanning. The input excitation laser beam was linearly polarized by a combination between an achromatic quarter-wave plate (AQWP05M-980, Thorlabs) and an achromatic half-wave plate (AHWP05M-980, Thorlabs) mounted in motorized rotation stages (PRM1/MZ8, Thorlabs) and placed in the laser beam path before the microscope. A polarimeter (PAX1000IR1, Thorlabs) was used to check for the purity of the linearly polarized laser beam and an absolute ellipticity angle of less than 0.3° and power variations less than 7% were obtained. The SHG was collected in both forward and backward directions, while TPEF was simultaneously acquired with SHG in epidetection. A 40X magnification and 0.75 numerical aperture (NA) objective was used to focus the excitation laser beam on the samples and to collect the TPEF and the backward-generated SHG (BSHG). The spectrally resolved detection setup inherent to the Leica TCS SP was used for collecting the BSHG (430 to 440 nm) and the TPEF (450 to 700 nm) on two separate channels. The forward-generated SHG (FSHG) was collected by using a 0.9 NA condenser lens and was spectrally separated from the excitation beam and TPEF signals by a combination of a shortpass filter (FF01-750/SP-25, Semrock) and a bandpass filter (FB430-10, Thorlabs) placed on the forward detection path.

PSHG image stacks consisting in 10 polarization-dependent images were acquired by rotating the linear laser beam polarization by increments of 20° from 0° to 180°. For each selected ROI both the FSHG and BSHG image stacks were acquired. Simultaneously with the B/FSHG acquisition, the TPEF was also collected. All the images were collected by averaging 3 frames of 512 × 512 pixels acquired at 200 lines/s. Each scanned area size was determined based on previous results^28^. The optimal pixel size computed according to the Nyquist criterion, the requirement of field of views large enough to accommodate the presence of meaningful collagen information, and facile interpretation by a pathologist, were considered when choosing the scan size. According to these criteria, we included images of 125 × 125 µm^2^.

### Biophysical collagen model

The collagen model used in this work is essentially a single-axis molecule model which has been previously extensively described^26,35,36^. The model estimates the intensity of the SHG signal generated by a harmonophore (in our case collagen) depending on the excitation beam polarization orientation (α), on the in-plane orientation of the SHG active molecule (*φ*), and on the macroscopic nonlinear susceptibility tensor (χ^(2)^) assuming cylindrical symmetry for collagen along the main fibril axis and that the collagen fibril is lying in the imaging plane:

1 $${I}_{SHG}={I}_{0}\cdot \left[{{\sin }}^{2}\,2\left(\varphi -\alpha \right)+{\left(\frac{{\chi }_{31}}{{\chi }_{15}}\cdot {{\sin }}^{2}\left(\varphi -\alpha \right)+\frac{{\chi }_{33}}{{\chi }_{15}}\cdot {{\cos }}^{2}\left(\varphi -\alpha \right)\right)}^{2}\right]$$

where I_0_ is a multiplication factor depending on the excitation intensity and the χ^(2)^ tensor element used for normalization (in this case χ_15_), and χ_15_, χ_31_, χ_33_ are the only nonzero elements of the χ^(2)^ tensor under the assumption of cylindrical symmetry of collagen.

Another form of the model expressed in (1) was found to be more suitable for fitting the experimental PSHG images using a Fourier series expansion:

2 $${I}_{SHG}={I}_{0}\cdot \left[{c}_{0}+{c}_{1}\cos \left(2\left(\varphi -\alpha \right)\right)+{c}_{2}\cos \left(4\left(\varphi -\alpha \right)\right)\right]$$

Here the coefficients c_0_, c_1_ and c_2_ are related to the parameters χ_15_, χ_31_, χ_33_^7,37^. This approach was proposed for a Fast Fourier Polarization SHG analysis (FF-PSHG)^7^ which retrieves the biophysical parameters of the proposed model in a fast and reliable procedure. The variation of the SHG intensity with the excitation laser beam polarization angle is processed in the Fourier space by computing the discrete Fourier Transform (DFT) coefficients (F_i_) for the polarization dependent intensity for each pixel in the acquired images. In our case, the PSHG image stack is acquired with a polarization angle from 0° to 180° in steps of 20°, resulting in a total of 10 images. Since in a polarization experiment the period is 180°, images at 0° and 180° should be identical and can be used to check the system stability during the PSHG image stack acquisition. We average the two images, hence the stack which is used for processing consists in 9 images. For each pixel in the PSHG stack the DFT coefficients F_i_ are computed and are further used to determine the Fourier series coefficients c_i_ in each pixel. The coefficients c_i_ are then used to calculate the χ^(2)^ tensor elements ratios χ_31_/χ_15_, χ_33_/χ_15_, χ_33_/χ_31_^7,37^.

The FF-PSHG analysis offers the possibility to calculate two parameters related to the molecular structure of the SHG active assembly: the in-plane orientation of collagen (*φ*)^7^, and the orientation of the hyperpolarizability tensor dominant axis (θ_*e*_), which was previously related to the helical pitch angle of the collagen triple helix. The helical pitch angle can be calculated from the already known parameters as follows^26^:

3 $${\theta }_{e}=\frac{{\chi }_{33}/{\chi }_{15}}{2+{\chi }_{33}/{\chi }_{15}}$$

Hence, the FF-PSHG analysis performed on a PSHG stack retrieves 5 images corresponding to the pixel-level calculation of each of the following parameters: χ_31_/χ_15_, χ_33_/χ_15_, χ_33_/χ_31_, *φ* and θ_*e*_.

These parameters were previously used for quantitatively assessing the status of the collagen architecture (Table 1). For example, we have previously used χ_31_/χ_15_ and χ_33_/χ_15_ ratios extracted from PSHG image stacks acquired on the collagenous capsule surrounding thyroid nodules^9^ as a differential diagnosis marker for encapsulated malignant and benign thyroid nodules. The same structural information on collagen was used to assess the influence of the H&E staining on the outputs of typical quantitative analyses of PSHG imaging^38^.

**Table 1:** A summary of the PSHG microscopy parameters extracted from the biophysical collagen model used for quantifying the collagen structure in different tissues and pathologies.

Tissue type & pathology	PSHG parameters
Mouse & rat liver fibrosis	χ_33_/χ_15_^66,67^, χ_31_/χ_15_^67^
Melanoma in mice	*φ*^68^, χ_33_/χ_15_^68^
Equine meniscus	*φ*^69^
Tendons	χ_33_/χ_15_^70,71^
Skin	*φ*^72^
Cornea	*φ*^73,74^, χ_33_/χ_15_^74^
Pulmonary fibrosis	θ_*e*_^60^
Esophageal squamous cell carcinoma	χ_33_/χ_15_^75^
Ovarian cancer	θ_*e*_^59^
Breast cancer	χ_33_/χ_15_^55,76^, χ_33_/χ_31_^55^

### Fitting quality estimators

Different strategies can be used to evaluate the quality of the fitting procedures. Pixels with erroneous results can be selected by the coefficient of determination (R^2^). We have considered the most general definition for the coefficient of determination:

4 $${R}^{2}=1-\frac{sum\,of\,squared\,residuals}{total\,sum\,of\,squares}$$

In our case, the dataset consists in 10 values for every given pixel depending on the input laser polarization angles (0° to 180° in steps of 20°), hence taking the form of a vector **X** = [x_1_ x_2_ … x_10_], while the fitted or predicted data is also a vector with SHG intensity values **Y** = [y_1_ y_2_ … y_10_], where each element is computed with Eq. 1, with the values for χ_31_/χ_15_, χ_33_/χ_15_ and *φ* being obtained from the fitting algorithm. The sum of squared residuals (SSR) is then: $SSR={\sum }_{i=1}^{10}{\left({x}_{i}-{y}_{i}\right)}^{2}$ and the total sum of squares (SST) is $SST={\sum }_{i=1}^{10}{\left({x}_{i}-\bar{x}\right)}^{2}$, with $\bar{x}$ being the mean of the input data.

Another approach considers the fact that only coefficients c_0_, c_1_ and c_2_ have biophysical meaning according to the model in Eq. 2. Hence the other coefficients which can be calculated based on the DFT coefficients (c_3_ to c_8_) are considered as noise^37^ since they do not contribute to the calculation of PSHG parameters. Therefore, the experimental error in determining the spectral components with biophysical significance can be expressed as^7^:

5 $$ERR=\frac{mean\left({c}_{3}\,to\,{c}_{8}\right)}{mean\left({c}_{0},\,{c}_{1},\,{c}_{2}\right)}$$

Pixels in areas outside any SHG-active tissue is noise and therefore results in a value of ERR closer to unity, while pixels in areas with a good signal to noise ratio will result in ERR *≈* 0.

Based on the previous assumptions, a signal-to-noise ratio (SNR) can be calculated for each pixel considering that a power spectrum can be computed from the DFT, with the signal power corresponding to each DFT component being given by:

6 $${P}_{{F}_{i}}={\left|{F}_{i}\right|}^{2}$$

where F_i_ is a spectral DFT component, whose index *i* ranges from 1 to 9 since 9 different polarizations were used from the image stack in the FF-PSHG analysis.

Due to the symmetrical nature of the Fourier Transform and evaluating the noise by considering the spectral Fourier components that have no biophysical meaning and contribution to the model^39^, the SNR was calculated as:

7 $$SN{R}_{dB}=1{0\log }_{10}\frac{{\sum }_{i=1}^{2}{\left|{F}_{i}\right|}^{2}}{{\sum }_{i=3}^{6}{\left|{F}_{i}\right|}^{2}}$$

Apart from the 5 images with biophysical significance retrieved by the FF-PSHG analysis, three additional images are provided, representing R^2^, ERR and SNR. These can be used to filter out pixels that may be regarded as being unsuitable for further analysis.

### Local dispersion estimators

We have considered three measures of dispersion and randomness which can be used to characterize the texture of the images obtained by the FF-PSHG analysis.

The entropy of a variate X having *n* possible values (x_1_, x_2_, …. x_n_) and for each value corresponding the probability p(x_1_), p(x_2_), …p(x_n_) is defined as follows:

8 $$ENT(X)=-{\sum }_{i=1}^{n}\,p({x}_{i})\cdot {\log }_{2}[p({x}_{i})]$$

In the case of an image, the discrete variate X elements are the pixel values, with p(x_i_) being the occurrence rate of a pixel value x_i_. Entropy (ENT) was previously used to characterize the distribution of contrast levels in SHG images for the collagen architecture characterization in mice prostate^40^, to assess SHG image quality^41^ or to characterize the microstructure of foetal membrane and its response to deformation^42^.

Standard deviation (SD) and median absolute deviation (MAD) are both measures of statistical dispersion of a set of values. While SD is a statistic that measures the dispersion of a dataset relative to its mean, MAD is defined as the median of the absolute deviations from the median and is less sensitive to outliers than SD^43^. If the dataset is normally distributed, then SD is the better choice for assessing spread, while for situations with data which is not normally distributed, MAD is the more robust measure to use. Both SD and MAD have been previously used for SHG image analysis^9,44^.

**Fig. 2:**
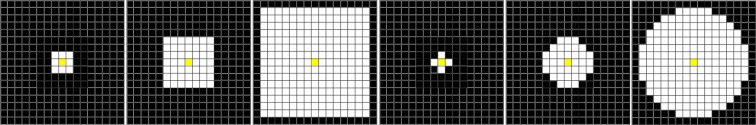
Square and “circular” windows used for local dispersion estimation. These computational windows with the side (diameter) of 3, 7 and 15 pixels, respectively were used for local ENT, MAD and SD calculation, with the resulting value corresponding to the center of the window (yellow pixel) in the new generated image.

In the proposed image collection, ENT, MAD and SD were used to generate new images starting from the images with biophysical significance retrieved by the FF-PSHG analysis (χ_31_/χ_15_, χ_33_/χ_15_, χ_33_/χ_31_, *φ* and θ_*e*_) by locally calculating the entropy, median absolute deviation and standard deviation, respectively on two types of computational windows. We have considered a square window with the side of 3, 7 and 15 pixels, and a “circular” window with the same diameters (Fig. 2).

With respect to the usefulness of these representations, we find noteworthy to recall that we have previously used SD and MAD locally computed on collagen orientation images^9^ to assess the pixel level angular distribution of collagen in thyroid nodule capsules with application in differential diagnosis of encapsulated thyroid nodules.

A custom created Matlab code and six FIJI macros^45^ were used to compute from the raw PSHG image sets the collagen structure-related images, the fitting quality estimator images and the local dispersion images using ENT, MAD and SD on square and “circular” windows.

## Data Records

The image collection, PSHG-TISS^46^, is comprised of PSHG image sets acquired on breast, skin and thyroid tissue samples (Table 2). The OSF record^46^ includes beside the PSHG image sets additional computationally generated images which can be used to complement the subjective qualitative analysis of second harmonic generation (SHG) images. These latter have been calculated using the single-axis molecule model for collagen and provide 2D representations of different specific PSHG parameters known to account for the collagen structure and distribution. The structure of the OSF record^46^ which includes the PSHG-TISS image collection and the image files is described in the following.

**Table 2:** Tissue types and ROIs classification configurations addressed in the proposed PSHG image collection.

Tissue section type	ROI classification	ROI ID
Breast, unstained	normal TDLU	breast_1 to breast_18
	ductal carcinoma *in situ*	breast_19 to breast_30
	invasive carcinoma	breast_31 to breast_48
Breast, H&E-stained	normal TDLU	breast_HE_1 to breast_HE_18
	*in-situ* carcinoma	breast_HE_19 to breast_HE_30
	invasive carcinoma	breast_HE_31 to breast_HE_47
Skin, unstained	papillary dermis	skin_1 to skin_10
	reticular dermis	skin_11 to skin_32
	subcutaneous tissue	skin_33 to skin_53
Skin, H&E-stained	papillary dermis	skin_HE_1 to skin_HE_10
	reticular dermis	skin_HE_11 to skin_HE_32
	subcutaneous tissue	skin_HE_33 to skin_HE_53
Thyroid, H&E-stained	papillary thyroid carcinoma	thyroid_1 to thyroid_20
	follicular adenoma	thyroid_21 to thyroid_40

Images on breast tissue samples were acquired on three ROI classes. The first refers to normal terminal ductal-lobular units (TDLUs), which are the basic functional ductal/glandular structures of the breast^47^. The second ROI class corresponds to ductal carcinoma *in situ* (DCIS) which is considered the earliest form of breast cancer, recognized by intraductal atypical epithelial proliferations^48^. The third acquired breast ROI class is the invasive breast carcinoma of no special type which is also commonly known as invasive ductal carcinoma not otherwise specified and comprises the most frequent type of invasive breast cancers^49^. It is called “no special type” since it is morphologically heterogeneous and does not exhibit any specific features.

Images on skin samples were acquired on three distinct regions within the skin: papillary dermis, reticular dermis, and subcutaneous tissue. The papillary dermis^50^ is the upper portion of the dermis beneath the epidermis, characterised by randomly arranged collagen fibers and thin elastic fibers. The deep portion is the reticular dermis, which extends from the base of the papillary dermis to the surface of the subcutaneous tissue and is composed of coarse elastic fibers and thick collagen bundles parallel to the skin surface. The subcutaneous tissue, subcutis or hypodermis is the deepest layer of the skin and contains lobules of mature adipocytes surrounded by thin connective tissue septa which were captured in the SHG images in this collection.

Images on thyroid samples were acquired on the collagenous capsules surrounding thyroid nodules diagnosed as either follicular adenoma or papillary thyroid carcinoma. Follicular adenoma^51^ is a benign encapsulated thyroid tumor that shows evidence of follicular differentiation but lacks evidence of infiltrative growth pattern (capsular and/or vascular invasion). Papillary thyroid carcinoma^52^ is the most common endocrine malignancy. These solitary or multifocal lesions present follicular cell differentiation with characteristic distinctive nuclear features and with papillary, solid, trabecular, or follicular architecture and might be encapsulated.

For unstained tissue sections PSHG image stacks were acquired on 48 ROIs for breast samples, on 53 ROIs for skin samples and on 40 ROIs for thyroid samples. For the case of H&E-stained tissue sections, for breast and skin sections PSHG image stacks were acquired on 47 and 53 ROIs, respectively. For each ROI, PSHG image stacks were simultaneously acquired for both FSHG and BSHG detection configurations.

The proposed image collection, PSHG-TISS^46^, is thus comprised of 482 PSHG image stacks each consisting in 10 images acquired at varying linear laser beam polarizations (from 0° to 180° in steps of 20°). The as-acquired SHG images can be found in folders entitled [tissue type]_[ROI number] with two subfolders BSHG and FSHG corresponding for the two detection configurations. SHG images in these folders are named under the following nomenclature: [tissue type]_[HE]_[ROI number]_[detection configuration]_[polarization angle]. Each of the ROI folders include a TPEF image under the following name: [tissue type]_[HE]_[ROI number]_TPEF. The TPEF image can be used to place the collagen imaged by SHG microscopy in a tissular context. For the case of unstained tissue sections [HE] is missing in the file name for both SHG and TPEF images. All the SHG images in both detection configurations and the TPEF image are 8-bit images and are made available in .tif file format. They can be opened with any image viewer/processing software, e.g., the freeware image viewer IrfanView or ImageJ^53^. When opened under ImageJ, the TPEF images have already an automated contrast improvement set. Each of the FSHG and BSHG folders contains a *Results* subfolder including computationally generated images using the FF-PSHG fitting algorithm and images for local dispersion for the structural collagen properties. Images resulting directly from the FF-PSHG fitting algorithm are named corresponding to the computed parameter discussed in the *Biophysical collagen model* (CHI3115, CHI3315, CHI3331, FI and THETA) and *Fitting quality estimators* (R2, SNR and ERR) subsections (Table 3). Images computed using the local dispersion approaches, described in the *Local dispersion estimators* subsections, use the following nomenclature: [collagen structural property parameter]_[local dispersion estimator]_[type of computational window]_[dimension of the computational window]. All the computationally generated images are given as 32-bit images available in.tif file format and can be opened using ImageJ.

**Table 3:** Correspondence between the file names for the computationally generated images and the FF-PSHG analysis parameter.

File name	CHI3115	CHI3315	CHI3331	FI	THETA	R2	SNR	ERR
FF-PSHG parameter	χ_31_/χ_15_	χ_33_/χ_15_	χ_33_/χ_31_	*φ*	θ_*e*_	R^2^	SNR	ERR

In brief, in the proposed PSHG image collection^46^ the titles of the root folders represent the tissue type either unstained or H&E-stained, the titles of level one folders represent the individual imaged ROIs, the titles of level two folders represent one of the SHG acquisition configurations, while the titles of level three folders represent results from the FF-PSHG algorithm and the local dispersion images for structural collagen parameters. The image collection consists in a total of 52,297 images divided as follows: 4,820 SHG images, 241 TPEF images, 3,856 images resulting from the FF-PSHG algorithm and 43,380 images of local dispersion for collagen structure parameters.

## Technical Validation

The proposed PSHG image collection contains raw PSHG image stacks acquired on three thin tissue types: breast, skin and thyroid. Using the PSHG image stacks and the FF-PSHG analysis the image collection was enlarged with the outputs of the fitting algorithm described in the Methods section for each of the PSHG image stacks. The images obtained from the FF-PSHG analysis with collagen structural significance were further used to compute images of local distribution of the parameters.

**Fig. 3:**
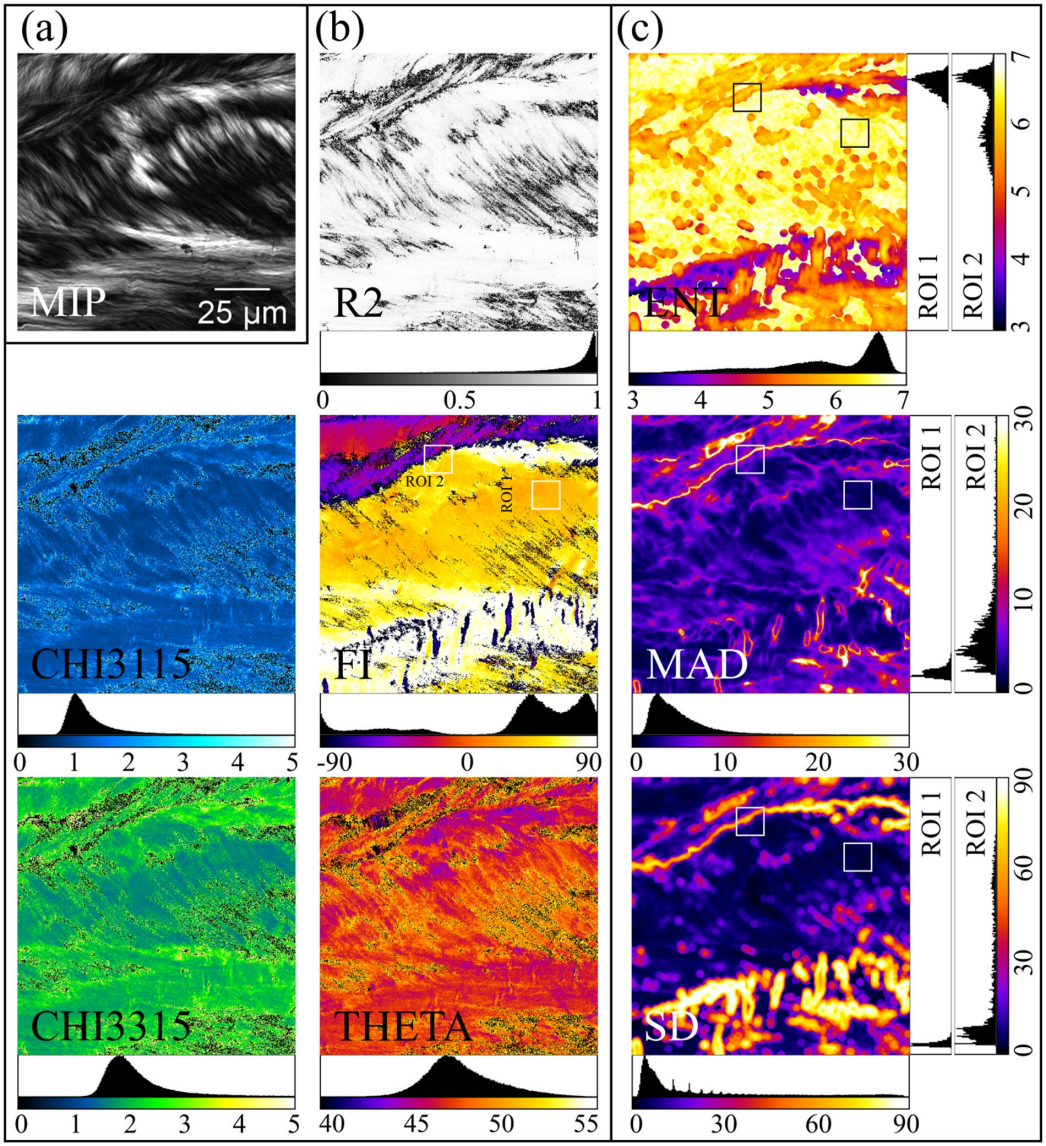
Images obtained from a PSHG image stack acquired on the thyroid capsule. (**a**) The entire PSHG stack is represented here as the maximum intensity projection (MIP). (**b**) Images obtained using the FFT-PSHG analysis: a measure of the fitting quality (R2 image) and images with collagen structural information (CHI3115, CHI3315, FI, THETA. (**c**) Local dispersion images (ENT, MAD and SD) for the FI image displayed here are calculated using a circular computational window with a diameter of 15 pixels. Histograms are provided for all computed individual images as well as for two ROIs selected from the FI image and the local dispersion images, respectively.

To technically validate the outputs of the FF-PSHG analysis, we refer to the reliability of the obtained results. The R2 image in Fig. 3, for example, demonstrates a value for R^2^ close to unity which is aligned with a good fit of the experimental data with the theoretical collagen model. Considering a 0.8 threshold for R^2^ (as we have used in our previous publications^23,38^), 83% of the pixels qualify as a good fit, while a more restrictive threshold for R^2^, 0.9, as used by other groups^35^, still includes more than 68% of the pixels.

Computed values reflecting the structural collagen properties are also aligned with those previously reported in the literature. For the χ_31_/χ_15_ ratio we obtained values close to unity (CHI3115 image and corresponding histogram in Fig. 3) which confirms the validity of Kleinman symmetry^15^ for an excitation wavelength of 870 nm. For the case of χ_33_/χ_15_ (CHI3315 image and corresponding histogram in Fig. 3), the mode value is 1.79 which is in good agreement with previous results obtained on human tissue samples (e.g., lung^54^, breast^55^ or skin^56^). Other publications report higher values for χ_33_/χ_15_, but consider the mean value obtained for thyroid^57^ or pancreas^58^ tissue samples which is comparable with our results (the mean value for χ_33_/χ_15_ is 2.15). The observed mode value in the histogram for the helical pitch angle of 46.7° (THETA image and the corresponding histogram in Fig. 3) is consistent with previous studies on human ovary^59^ or lung^60^ tissue and with values reported^26,61^ to be obtained for the angles of the collagen helix relative to the main filament axis (~45°) as obtained from X-ray diffraction data.

The images obtained by applying the local estimators for dispersion or randomness in round/square computational windows were computed from images obtained from the FF-PSHG analysis which provide collagen structural information. Images obtained from the collagen orientation image (FI in Fig. 3) for a circular window with a diameter of 15 pixels are exemplified in Fig. 3 (images ENT, MAD and SD). Two ROIs were selected in the FI image: ROI 1 on an area with a narrow distribution for collagen orientation and ROI 2 which is representative for a broad distribution. The histograms on ENT, MAD and SD images (Fig. 3) for the selected ROIs demonstrate a potential of the dispersion/randomness local estimators to assess the local collagen orientation. The histograms in the case of MAD and SD images for the two ROIs indicate, as expected, increased values for the broader distribution of collagen orientation compared to the narrower distribution. On the other hand, for the ENT image, a broader distribution of the collagen orientation is translated into lower values of entropy (histograms corresponding to ROI 1 and 2 for the ENT image in Fig. 3).

## Usage Notes

### Exploring different collagen fitting approaches

To date, different collagen models were used to fit the PSHG collagen experimental data (i.e., the single-axis molecule model^7^, the generic model^8^). Such collagen models assume a cylindrical symmetry of the collagen fibril which in some experiments has demonstrated its limitation, with a trigonal symmetry being considered more appropriate for fitting the experimental data^55,62^. The PSHG-TISS dataset can be used for the development and testing of more general collagen models supported by fast algorithm like the FF-PSHG analysis or the linear least square fitting method which can be extended to the case of complex molecular orientation distribution^63^. Such approaches might enable a better fitting of the experimental PSHG datasets with better results in identifying collagen signatures related to tissue pathologies.

### Intensity SHG images and TPEF images

The PSHG-TISS dataset can be used to support the development of image processing, computer vision or machine learning methods focused on SHG intensity images. To this end, each acquired PSHG image stack can be used to generate a polarization independent image using different strategies. For example, all 10 images or only three^64^ of them acquired at different linear laser beam polarizations with a polarization angle step of 60°, can be averaged to result in a single representation that is polarization independent. Other methods such as the maximum intensity projection (MIP), or the smooth 2D manifold extraction^65^ can be used to obtain single representations harbouring information from the entire stack. Furthermore, PSHG-TISS can support the development of other methods aimed at similar purposes.

The SHG polarization independent images can either be used to develop image processing or analysis methods that focus at SHG signals alone, or those addressing composite SHG + TPEF images^12^. In PSHG-TISS, TPEF images were acquired on unstained and H&E-stained tissue sections. For unstained tissues, the endogenous autofluorescence sources were responsible for the recorded TPEF signals: metabolic substrates (e.g., NADH and FAD), structural proteins (e.g., elastin and keratin), lipofuscins, and melanin. In the case of the H&E-stained tissue sections, the TPEF signals originate from both the endogenous fluorophores as well as from the eosin-stained structures. Usually, eosin marks proteins non-specifically within the cytoplasm, borders of the cell membrane, red blood cells and extracellular structures (including collagen). The availability of TPEF images collected on both stained and unstained tissues can potentially support the development of image processing methods that can digitally separate between endogenous and exogenous fluorescence. Such methods would be very useful to promote new applications for TPEF imaging of stained tissues, which at present are not considered to accompany SHG imaging of stained tissues, as the contribution of exogenous fluorescent signals is believed to hamper the tissue intrinsic autofluorescence, which is the main focus of most TPEF tissue imaging applications.

### Collagen segmentation

We envision that the proposed image collection can be useful in the development and benchmarking of image processing and analysis methods relevant for SHG images such as image thresholding using different parameters for selecting relevant pixels. Three fitting quality estimators accompany the images created by running the FF-PSHG analysis: R^2^, SNR and ERR. All were previously used^7,28,39^ to select relevant pixels for further display or processing, but only R^2^ values were used for collagen segmentation in determining the fitting efficiency^28^. Extending the concept of fitting efficiency by using different strategies based on other fitting quality estimators might extend its use to determining the most appropriate collagen model for different types of tissues.

### Classification experiments

The potential usefulness of this PSHG image collection is augmented by the fact that it is accompanied by annotations (Table 2) regarding either the pathology (for breast and thyroid tissue samples) or the tissue region (for skin samples) from where the ROIs were acquired. All these information enable the utilization of the proposed PSHG image collection in the development of data augmentation strategies or transfer learning approaches which might be required for deep learning approaches. The mentioned strategies may be useful to overcome the current deep learning limitation of needing extended image libraries. This is important as most often images acquired using different microscopy techniques, especially when dealing with human tissues, come in limited sets, unsuitable for DL approaches. We consider that using local dispersion/randomness strategies as those proposed in the current image collection might be useful for the augmentation of image sets used for classification under DL approaches. At present, most data augmentation strategies used in DL applications dealing with microscopy images are the same as those used in DL applications focusing on natural images, e.g., flipping, rotation, etc. While the usefulness of these data augmentation schemes cannot be neglected, other, more specific augmentation methods would be of great benefit. In the context of PSHG imaging, as shown in this paper, from a single PSHG stack, various types of representations can be assembled. All of them harbour complementary information that has been found useful to assess various aspects of the collagen architecture. We hypothesize that training a neural network on these types of representations (that can be thought as data augmentation methods), in addition to training it on the raw PSHG stack, can enrich the neural network with additional knowledge and subsequent data analysis capabilities. This is a subject that we plan to pursue in next work.

Last but not least, we believe that the proposed image collection can also play an important role in inspiring and supporting the development of transfer learning approaches, where training is efficiently done on synthetic image sets that have been computationally generated, to result in frameworks that can accurately classify real (P)SHG images which are obviously harder to experimentally acquire in large numbers. To our knowledge, the use of collagen structural parameters extracted from PSHG image stacks is currently not explored in corroboration with DL strategies, and hope that this data set and the discussions presented here will inspire such efforts.

## Data Availability

The Matlab code used for the PSHG image sets fitting with the single-axis molecule model for collagen and the FIJI macros used to generate the entire dataset are available at: 10.17605/OSF.IO/K2Z8G^45^.
